# Effects of Powdery Cellulose Nanofiber Addition on the Properties of Glass Ionomer Cement

**DOI:** 10.3390/ma12193077

**Published:** 2019-09-20

**Authors:** Takako Nishimura, Yukari Shinonaga, Chikoto Nagaishi, Rie Imataki, Michiko Takemura, Keiichi Kagami, Yoko Abe, Kyoko Harada, Kenji Arita

**Affiliations:** 1 Department of Pediatric Dentistry, School of Dentistry, Osaka Dental University, 8-1, Kuzuhahanazono-cho, Hirakata-shi, 573-1121 Osaka, Japan; nisimura@cc.osaka-dent.ac.jp (T.N.); abe-y@cc.osaka-dent.ac.jp (Y.A.); kyoko-w@cc.osaka-dent.ac.jp (K.H.); arita-k@cc.osaka-dent.ac.jp (K.A.); 2 Department of Pediatric Dentistry, Graduate School of Dentistry, Osaka Dental University, 8-1, Kuzuhahanazono-cho, Hirakata-shi, 573-1121 Osaka, Japan; chikoto-n@cc.osaka-dent.ac.jp (C.N.); imataki-r@cc.osaka-dent.ac.jp (R.I.); takemura-m@cc.osaka-dent.ac.jp (M.T.); kagami-k@cc.osaka-dent.ac.jp (K.K.)

**Keywords:** glass ionomer cement, cellulose nanofiber, flexural strength, compressive strength, diametral tensile strength, fluoride-ion release

## Abstract

In this study, we aimed to evaluate the effect of the addition of powdery cellulose nanofibers (CNFs) on the mechanical properties of glass ionomer cement (GIC) without negatively affecting its chemical properties. Commercial GIC was reinforced with powdery CNFs (2–8 wt.%) and characterized in terms of flexural strength, compressive strength, diametral tensile strength, and fluoride-ion release properties. Powdery CNFs and samples subjected to flexural strength testing were observed via scanning electron microscopy. CNF incorporation was found to significantly improve the flexural, compressive, and diametral tensile strengths of GIC, and the corresponding composite was shown to contain fibrillar aggregates of nanofibers interspersed in the GIC matrix. No significant differences in fluoride-ion release properties were observed between the control GIC and the CNF-GIC composite. Thus, powdery CNFs were concluded to be a promising GIC reinforcement agent.

## 1. Introduction

In dentistry, the increasing demand for direct filling materials compared to the use of traditional materials has been supported by changes in restorative techniques. Particularly for children, people with special needs, and those with dental fear, traditional drill-and-fill techniques are often unsuitable [1]. Since its invention in the early 1970s [2], glass polyalkenoate cement, also known as glass ionomer cement (GIC), composed of a mixture of fluoro-alumino-silicate glass powders and a polyacrylic acid solution, has been widely used as a restorative and preventive material in dental applications owing to its good adhesion to tooth structures, linear thermal expansion coefficient similar to that of dentin, biocompatibility, and fluoride-ion release and recharge capability [3,4,5,6]. However, compared to other restorative materials such as resin composites, amalgams, and metals, GIC exhibits low mechanical strength [4]. Although many researchers have attempted to address this problem through the addition of reinforcements [7,8,9,10], the widespread application of this strategy has been hindered by its numerous drawbacks.

Cellulose, a renewable and nature-abundant biopolymer, has found diverse applications in the fields of biomedicine, energy, environmental science, and water research [11]. In medical fields, cellulose is mostly used in the form of nanoparticles, i.e., cellulose nanofibers (CNFs) or cellulose nanocrystals (CNCs). In particular, CNFs are more than five times stronger than steel at a fifth of the weight, as cellulose molecular chains are stretched and are crystalline, while the corresponding coefficient of linear thermal expansion is extremely small (<1/50th that of glass), and the elasticity modulus is essentially constant over the range of −200 to +200 °C [12]. Recent environmental issues and the need to establish a recycling-based and sustainable society have primarily driven the fundamental research and application of CNFs [13].

CNFs are generally used as low-concentration watery suspensions. However, as the physical properties of most dental restorative materials deteriorate in the presence of water, supplementation of GIC with high loadings of CNFs (and consequently of water) is not practically useful. Recently, powdery CNFs have been developed to compensate for the disadvantages of CNF aqueous suspensions.

In this study, we aimed to modify a conventional GIC with water-free powdery CNFs and evaluate the effect of this modification on the flexural strength, compressive strength, diametral tensile strength, and fluoride-ion release properties of the obtained composite. The tested null hypotheses of our study are as follows: I) no difference is found between the mechanical strength of GIC and CNF-containing GIC; and II) no difference is found between the fluoride-ion release property of GIC and CNF-containing GIC.

## 2. Materials and Methods

### 2.1. Sample Preparation

A conventional GIC for pit and fissure sealing and tooth surface protection (Fuji VII^®^, GC Co., Ltd., Tokyo, Japan) was used. Both Fuji VII glass powder and Fuji VII liquid, containing polyacrylic acid, polybasic carboxylic acid, and water, were used in all control and experimental groups. Powdery CNFs (Cellulostar, STARLITE Co., Ltd., Osaka, Japan: STAR) were added to Fuji VII during mixing at a powder/liquid ratio of 1.8 according to the manufacturer’s recommendations to achieve loadings of 2–8 wt.%.

### 2.2. Mechanical Strength Evaluation

#### 2.2.1. Flexural Strength Test

For flexural strength tests, beam-shaped samples (*n* = 6/group) measuring 25 mm × 2 mm × 2 mm were prepared in a stainless steel split mold following the procedures outlined in ISO9917-2:2017 [14]. The cement was inserted into the mold with a syringe, covered with polyester strips at the top and bottom surfaces, and compressed using a glass plate with a load of 500 g for 10 min. The samples were stored at 37 °C and a relative humidity of 100% for 50 min, carefully removed from the molds, further stored in artificial saliva (Saliveht^TM^ Aerosol, Teijin Ltd., Osaka, Japan) for 23 h at 37 °C, and subjected to a three-point bending test using a universal testing machine (AGS-X, Shimadzu Corp., Kyoto, Japan) at a crosshead speed of 0.5 mm/min.

#### 2.2.2. Compressive Strength Test

For the compressive test, cylindrical samples (*n* = 6/group) measuring 4 mm in diameter × 6 mm in height were prepared in the stainless steel split mold following the procedures outlined in ISO9917-1:2017 [15]. The samples were carefully removed from the molds and further stored for 23 h in artificial saliva, as described above. Compressive strength tests were performed using a universal testing machine (AGS-X, Shimadzu Corp., Kyoto, Japan) at a crosshead speed of 1 mm/min.

#### 2.2.3. Diametral Tensile Strength Test

Cylindrical samples (*n* = 6/group) measuring 4 mm in diameter × 6 mm in height were prepared using the stainless steel split mold, stored in artificial saliva as described above, and subjected to a diametral tensile strength test. The test was performed using a universal testing machine (AGS-X, Shimadzu Corp., Kyoto, Japan) at a crosshead speed of 0.5 mm/min.

### 2.3. Scanning Electron Microscopy (SEM) Observations

Representative STAR samples and samples after the flexural bending test were coated with a thin layer of Osmium using a plasma Os coater (HPC-20, Vacuum Device Co., Ltd., Ibaraki, Japan) and then observed by SEM (S-4800, Hitachi High-Technologies Co., Tokyo, Japan).

### 2.4. Measurement of the Fluoride-Ion Release Dose

For fluoride-ion release measurements, specimens measuring 10 mm in diameter × 2 mm in thickness were prepared using a polyethylene split mold (*n* = 6/group), individually suspended in deionized water (8 mL) in sealed containers, and stored at 37 °C. Each disk was removed from water, washed with deionized water (2 mL), dried on filter paper, and immediately immersed into fresh deionized water (8 mL) for further measurement. Fluoride-ion concentrations were measured every day for seven days using an F ion–selective electrode (6561-10c, HORIBA Ltd., Kyoto, Japan) connected to an ion meter (D-53, HORIBA, Kyoto, Japan).

### 2.5. Statistical Analysis

Data were presented in the form of mean ± standard deviation (S.D.) and analyzed via the *t*-test or one-way ANOVA and Tukey’s test (KaleidaGraph 4.00, SYNERGY SOFTWARE, Reading, PA, USA), with *p* < 0.05 indicating statistically significant results. The confidence interval was set at 95%.

## 3. Results

Table 1 summarizes the mean flexural, compressive, and diametral tensile strengths (with the corresponding S.D.s) obtained for the control and STAR groups. The flexural strengths of 2, 4, 6 and 8 wt.% STAR-containing GICs significantly exceeded that of the control GIC (*t*-test, *p* < 0.05). Similarly, the compressive strengths of 4, 6 and 8 wt.% STAR-containing GICs significantly exceeded that of the control GIC (*t*-test, *p* < 0.05). Finally, the diametral tensile strengths of 6 and 8 wt.% STAR-containing GICs significantly exceeded that of the control GIC (*t*-test, *p* < 0.05). Notably, STAR loading had no significant effects on the flexural, compressive, or diametral tensile strengths (Tukey’s test, *p* > 0.05).

**Figure 1:**
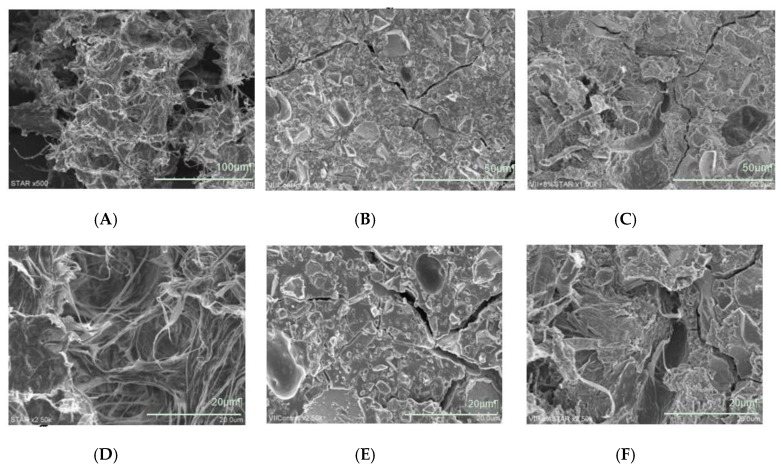
SEM images of STAR ((**A**) ×500 and (**D**) ×2500), control GIC ((**B**) ×1000 and (**E**) ×2500) and 8% STAR-containing GIC ((**C**) ×1000 and (**F**) ×2500) samples.

Figure 1 presents representative SEM images of STAR, control GIC, and 8 wt.% STAR-containing GIC samples after the flexural strength test, revealing the presence of innumerable irregular STAR fiber aggregates with a net-like structure (Figure 1A,D). The average aggregate had a size of ~100 µm and contained multiple air layers. Compared to the control GIC (Figure 1B,E), the 8 wt.% STAR-containing GIC featured numerous fibrous aggregates in its matrix layer (Figure 1C,F). The GIC matrix penetrated the STAR aggregate, and the original pre-addition form could be observed only in parts of aggregate tips.

**Figure 2:**
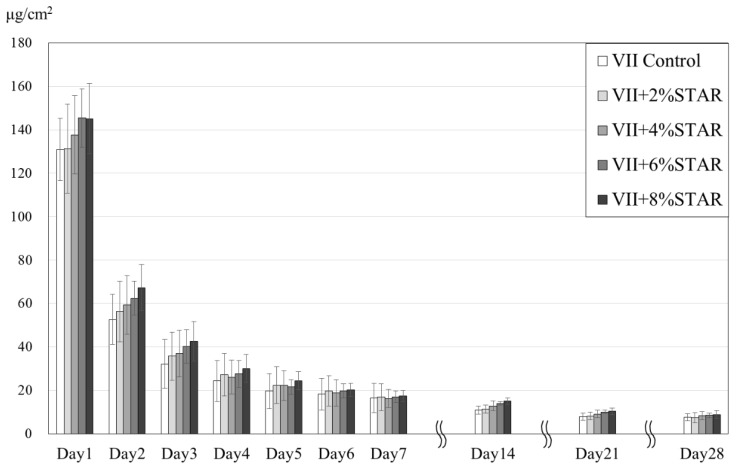
Amounts of fluoride ions released from control and STAR-containing GICs. There are no significant differences between all group at each period (ANOVA/Tukey; *p* > 0.05).

As shown in Figure 2, the fluoride release amount increased with increasing CNF loading. However, no significant differences were observed between the control GIC and STAR-containing GICs, and between GICs with various CNF loadings (*p* > 0.05).

## 4. Discussion

Previous studies [16,17,18] have suggested that CNC addition can increase the compressive strength of GIC. The three (flexural, compressive, and diametral tensile) mechanical strength tests performed herein demonstrate that the addition of powdery CNFs improves the compressive, flexural, and diametral tensile strengths of GIC, which is a brittle material featuring a tensile strength markedly lower than its compressive strength. The flexural strength of a given material represents its ability to bend before it breaks and is attained when the ultimate flexibility is reached before its proportional limit. Restorative materials should exhibit high flexural strengths as they are exposed to chewing stress that might induce permanent deformation [10]. Mount suggested that the weakness of GIC originates from its matrix, which is prone to crack propagation under load, particularly in the presence of in-matrix defects [4]. Herein, SEM imaging showed that despite some aggregation, CNFs were well dispersed in the GIC matrix. The improvement in mechanical strength was ascribed to the characteristics of CNF structure, namely to the low fibril width (several nanometers) and a wide range of fibril lengths (several micrometers). Xu et al. reported that at constant loading, the incorporation of CNFs into a polyethylene oxide matrix results in higher strength and modulus than in the case when CNCs are employed [19]. CNFs are obtained from cellulose via chemical and mechanical methods, whereas CNCs are obtained from macroscopic or microscopic forms of cellulose by hydrolysis with strong acids [11]. CNCs are needle-like cellulose crystals with a width of 10–20 nm and a length of several hundred nanometers, whereas CNFs form long, flexible fiber networks with a fibril diameter similar to or larger than that of CNCs. In view of their larger aspect ratio and fiber entanglement with the GIC matrix, CNFs were considered to be more effective for conventional GIC reinforcement than CNCs, as confirmed by SEM imaging (Figure 1C). Garoushi et al. reported that although the flexural strength of a conventional GIC increased after reinforcement with hollow discontinuous glass fibers, this reinforcement did not improve compressive strength [19]. Thus, the beneficial effect of powdery CNFs on both the compressive and flexural strength of the GIC was attributed to the presence of fibril net-like structures and CNF flexibility.

Cellulose microfibers (CmFs) or CNCs have been used to enhance the mechanical and chemical properties of conventional GICs [16,17,18]. The corresponding studies showed that CmF incorporation does not significantly improve GIC mechanical properties [16,17], while the addition of small amounts of CNCs (0.2–0.4 wt.%) considerably increases compressive and diametral tensile strengths and elastic modulus compared to values obtained after the addition of larger amounts of CNCs [17,18]. SEM imaging performed by Silva et al. did not provide any indication of the greater interaction of CmFs with the GIC matrix. In our previous study [20], the incorporation of micro-sized and rod-shaped or spherical cellulose particles into a conventional GIC did not significantly improve compressive strength, and it was suggested that the GIC matrix does not chemically react with cellulose particles. On the contrary, CNCs or CNFs are generally produced in the form of low-concentration aqueous dispersions, e.g., a ~10-mg/mL CNC aqueous suspension was used by Silva et al. [17]. As the amount of CNC fibrils increases, so does the amount of water, which reduces GIC strength. Therefore, the amount of CNC or CNF suspensions that can be added to a given GIC is limited, and we consequently considered the use of highly concentrated CNF aqueous suspensions or powdery CNFs. Moreover, the use of aqueous suspensions poses the problems of high transportation cost and bacterial contamination [21], encouraging studies on the drying of cellulose nanomaterials. During drying, CNFs become strongly aggregated due to hydrogen bonding, which inhibits their re-dispersion and reduces transparency and viscosity [22]. Thus, great care should be taken to prevent aggregation during the drying of CNF materials. Herein, we could supplement a conventional GIC with large amounts (4–8 wt.%) of fibrils, as STAR did not contain water, and demonstrated that powdery CNFs are an excellent GIC reinforcement. The employed powdery CNFs were prepared via sublimation of an aqueous CNF dispersion, and the fibril surfaces were hydroxylated. SEM imaging revealed that CNF fibrils were partially aggregated and not fully dispersed in the GIC matrix. However, it did reveal that the GIC matrix penetrated the STAR network structures. It was suggested that the hydrophilicity of powdery CNFs (STAR) accounted for their compatibility with the GIC matrix.

Fuji VII is broadly used for pit and fissure sealing, root surface protection, and intermediate restoration owing to its better fluoride-ion release properties compared to those of other Fuji GIC products [23]. These properties are extremely important as fluoride ions interfere with the metabolism of enamel-binding bacteria, thereby making enamel more resistant to acids while decreasing the extent of tooth demineralization [24]. In this study, we demonstrated that powdery CNFs do not inhibit fluoride-ion release from Fuji VII and showed that the amount of released fluoride ions increased with increasing CNF loading. As our previous work revealed that cellulose does not chemically react with GICs [20], the enhanced release of fluoride ions was ascribed to the powder-liquid ratio of Fuji VII, that is, aqueous polyacrylic acid solution could readily react with the fluoro-alumino-silicate glass of Fuji VII, the content of which was reduced by CNF introduction.

CNFs are quite thin compared with the wavelength of light (visible wavelengths: 400–800 nm) and have therefore attracted interest as a reinforcement for transparent resins [12]. Herein, the color tone of STAR-containing GIC specimens was found to be off-white, although no exact values were measured. To achieve high optical transparency, the cellulose fiber diameter must be smaller than the optical wavelength [25]. As the diameter of STAR fibrils did not fulfill this condition, GIC transparency decreased upon CNF introduction. 

The antibacterial property is one of the important factors for the prevention caries and secondary caries by GIC. In this study, we have not investigated the antibacterial activity of GIC containing CNFs. However, it was reported that adding fluorinated graphene to traditional GICs could improve their mechanical and tribological properties, but also improve their antibacterial properties [9]. CNF and graphene are the most advanced materials. One of the features of CNF is that it can maintain high transparency with less visible light wavelength absorption compared to carbon nanotubes and graphene, which are being investigated for use as nano-reinforcing materials. There are constant quests in the development of novel dental materials. One of the essential inquiries for biomedical materials is biocompatibility or toxicity, similar to antibacterial property. With regard to cellulose biocompatibility, different studies provide various results, which can be ascribed to the wide range of employed methodologies and sample preparation techniques. Unluckily, direct investigations on the biocompatibility of CNCs and CNFs are still rare [26]. As CNFs are naturally occurring materials built up from glucose subunits, they are considered to have a low environmental impact and high safety. Therefore, there are many studies related to the application of CNFs and CNCs to the biomedical field, such as wounding dressing, cartilage/bone regeneration, dental repairs, and cancer-curing drugs, and these reports were summarized by the review [27]. Future studies should pay more attention to the aspect of CNF/CNC safety, and we should verify and report the biocompatibility and toxicity of GIC containing CNF in future studies.

## 5. Conclusions

In this study, we aimed to modify a conventional GIC with water-free powdery CNFs. The addition of newly developed powdery CNFs to a conventional GIC allowed one to exclude the concomitant introduction of water and improved GIC flexural, compressive, and diametral tensile strengths without negatively affecting fluoride-ion release properties.

## Figures and Tables

**Table 1:** Results of flexural strength, compressive strength, and diametral tensile strength tests.

Group	Flexural Strength (MPa)		Compressive Strength (MPa)		Diametral Tensile Strength (MPa)	
	Mean (S.D.)	*p* ^1^	Mean (S.D.)	*p* ^1^	Mean (S.D.)	*p* ^1^
Control	16.36 (3.12)	–	102.26 (9.40)	–	10.61 (1.39)	–
+2%STAR	21.46 (3.84)	*	111.24 (10.34)	NS	11.36 (1.43)	NS
+4%STAR	24.13 (2.07)	***	119.59 (7.54)	***	12.28 (1.79)	NS
+6%STAR	23.72 (2.37)	***	114.43 (7.55)	*	13.59 (0.72)	***
+8%STAR	20.60 (2.19)	*	117.26 (3.52)	*	12.58 (1.18)	*

^1^*t*—test for control group. NS: no significant difference, * *p* < 0.05, ** *p* < 0.01, *** *p* < 0.001.
